# Two Polyhydroxyalkanoate Synthases from Distinct Classes from the Aromatic Degrader *Cupriavidus pinatubonensis* JMP134 Exhibit the Same Substrate Preference

**DOI:** 10.1371/journal.pone.0142332

**Published:** 2015-11-06

**Authors:** Xuan Jiang, Xi Luo, Ning-Yi Zhou

**Affiliations:** 1 Key Laboratory of Agricultural and Environmental Microbiology, Wuhan Institute of Virology, Chinese Academy of Sciences, Wuhan 430071, China; 2 State Key Laboratory of Microbial Metabolism & School of Life Sciences and Biotechnology, Shanghai Jiao Tong University, Shanghai 200240, China; University of Freiburg, GERMANY

## Abstract

*Cupriavidus pinatubonensis* JMP134 utilizes a variety of aromatic substrates as sole carbon sources, including *meta*-nitrophenol (MNP). Two polyhydroxyalkanoate (PHA) synthase genes, *phaC1* and *phaC2*, were annotated and categorized as class I and class II PHA synthase genes, respectively. In this study, both His-tagged purified PhaC1 and PhaC2 were shown to exhibit typical class I PHA synthase substrate specificity to make short-chain-length (SCL) PHA from 3-hydroxybutyryl-CoA and failed to make medium-chain-length (MCL) PHA from 3-hydroxyoctanoyl-CoA. The *phaC1* or *phaC2* deletion strain could also produce SCL PHA when grown in fructose or octanoate, but the double mutant of *phaC1* and *phaC2* lost this ability. The PhaC2 also exhibited substrate preference towards SCL substrates when expressed in *Pseudomonas aeruginosa* PAO1 *phaC* mutant strain. On the other hand, the transcriptional level of *phaC1* was 70-fold higher than that of *phaC2* in MNP-grown cells, but 240-fold lower in octanoate-grown cells. Further study demonstrated that only *phaC1* was involved in PHA synthesis in MNP-grown cells. These findings suggested that *phaC1* and *phaC2* genes were differentially regulated under different growth conditions in this strain. Within the *phaC2*-containing gene cluster, a single copy of PHA synthase gene was present clustering with genes encoding enzymes in the biosynthesis of PHA precursors. This is markedly different from the genetic organization of all other previously reported class II PHA synthase gene clusters and this cluster likely comes from a distinct evolutionary path.

## Introduction

Polyhydroxyalkanoate (PHA) is a class of biopolymer that is produced by a variety of microorganisms as carbon and energy storage components. It is usually synthesized when the carbon source is available in excess, and growth is limited by a lack of other essential nutrients [1, 2]. Based on the chain-length of the monomers for PHA synthesis, PHAs are typically categorized into two major types: short-chain-length (SCL) polymers (C3~C5 monomers) and medium-chain-length (MCL) polymers (C6 and longer) [3, 4]. The monomer types of PHA depend on the carbon sources, their metabolic routes and PHA synthases classes [1]. During PHA biosynthesis, PHA synthases are the key enzymes catalyzing the polymerization of hydroxyacyl-coenzyme A (CoA) thioesters concomitantly with the release of free CoA [3, 4]. Based on their substrate specificities and subunit compositions, PHA synthases have been categorized into four classes [3]. Class I and class II PHA synthases consist of a single type of subunit, while class III and IV synthases have two types of subunits. Class I, III and IV PHA synthases prefer substrates that are SCL hydroxyalkanoic acid CoA thioesters (C3~C5). In contrast, class II PHA synthases prefer MCL substrates (≥C6). Of the bacteria capable of producing PHA, some have more than one PHA synthase gene. These are usually homologs and belong to the same class of PHA synthases as reviewed by Bernd H. Rehm. [3]. Nevertheless, there are a few reported cases of bacterial strains carrying multiple functional PHA synthase homologs that come from different PHA synthase classes.

*Cupriavidus pinatubonensis* JMP134 (DSM4058, formerly known as *Cupriavidus necator* JMP134) is a versatile aromatic compounds degrader isolated from polluted environments. It has been deduced to contain two putative PHA synthase genes: *phaC1* (Reut_A1347) and *phaC2* (Reut_A2138) [5]. It has been predicted that *phaC1* encodes a class I PHA synthase, while *phaC2* encodes a class II PHA synthase [5]. However, the function of these two putative PHA synthase genes remains to be elucidated. On the other hand, strain JMP134 is capable of utilizing over 60 aromatic compounds as a sole carbon and energy source for growth, including the environmental pollutant *meta*-nitrophenol (MNP) [6–8]. Microbial MNP degradation has been given a great deal of attention in *C*. *pinatubonensis* JMP134. Its reductive catabolic pathway has been clearly illustrated at molecular and biochemical levels by Schenzle et al. [6] and our lab [9, 10]. However, the relationship between the aromatic degradation and PHA production has not yet been investigated in this strain, or any other aromatic utilizers. Research experience with the MNP degradation by strain JMP134 has naturally led us to explore the possibility of its PHA production when utilizing MNP as the carbon source.

In this study, we report that the *phaC1* and *phaC2* genes code for two functional PHA synthases in *C*. *pinatubonensis* JMP134 using genetic and biochemical approaches. Despite being classified into two classes based on their protein sequences, both these two PHA synthases exhibited activities towards SCL but not MCL substrate *in vitro*. Further, PHA can be produced when MNP was the sole carbon source *in vivo*, but only in the presence of *phaC1*. This not only provides us with a better understanding of the genetic and biochemical diversity of PHA synthesis in bacteria, but also reveals the link between PHA production and MNP degradation.

## Materials and Methods

### Bacterial strains and culture conditions

Bacterial strains and plasmids used in this study are listed in Table 1. Unless noted otherwise, all *Cupriavidus pinatubonensis* and *Pseudomonas aeruginosa* strains were grown at 30°C and shaken at 200 rpm in minimal salt medium (MSM) described previously [11] supplemented with 0.5 mM *meta-*nitrophenol or 2% (w/v) fructose or octanoate as the sole carbon source. All *E*. *coli* strains were grown in Lysogeny Broth (LB) medium at 37°C and shaken at 200 rpm. Antibiotics were added to the media at the following concentrations (μg·mL^-1^): chloramphenicol (Cm), 34; spectinomycin (Spec), 50; and tetracycline (Tc), 20.

**Table 1:** Bacterial strains and plasmids used in this study.

Strains or plasmids	Description or relevant genotype or phenotype^a^	Reference or source
**Strains**		
*Cupriavidus pinatubonensis*		
JMP134	Wild type strain, an aromatic degrader	[12]
JMP134Δ*phaC1*	*phaC1*-deleted mutant strain, Cm^r^	This study
JMP134Δ*phaC2*	*phaC2*-deleted mutant strain, Spec^r^	This study
JMP134Δ*phaC1*Δ*phaC2*	*phaC1* and *phaC2-*deleted mutant strain, Cm^r^ Spec^r^	This study
*Escherichia coli*		
DH5α	F− φ80(*lacZ*)ΔM15 Δ*lac*X74 *hsdR*(r_k_^−^m_k_^+^) Δ*recA*1398 *endA1 tonA*	TransGen Biotech, Beijing, China
WM3064	Donor strain for conjugation, 2,6-diaminopimelic acid auxotroph: *thrB1*004 *pro thi rpsL hsdS lacZ*ΔM15 RP4-1360 Δ(*araBAD*)567 *ΔdapA*1341::[*erm pir*(wt)]	[13]
BL21(DE3)	F− *ompT hsdS* (r_B_^−^m_B_^+^) *gal dcm*(DE3)	TransGen Biotech, Beijing, China
*Pseudomonas aeruginosa*		
PAO1	Wild type strain, MCL-PHA producer	[14]
PAO1Δ*phaC1*_*Pa*_ *-Z*_*Pa*_ *-C2*_*Pa*_	*phaC1*, *phaZ* and *phaC2*-deleted mutant strain, Cm^r^	This study
**Plasmids**		
pEX18Tc	Mobility verctor, *oriT* ^+^, *sacB* ^+^, Tc^r^	[15]
pEX18Tc-*cmgfp*	Chloramphenicol resistant gene and *gfp* gene cloned into pEX18Tc	[16]
pML300	A plasmid containing spectinomycin resistance gene *aadA*	[17]
pEX18Tc-*phaC1*cm	Derived from pEX18Tc for *phaC1* gene deletion, containing chloramphenicol resistance gene	This study
pEX18Tc-*phaC2*spec	Derived from pEX18Tc for *phaC2* gene deletion, containing spectinomycin resistance gene	This study
pEX18Tc-*pha*_*Pa*_cm	Derived from pEX18Tc for *phaC1*_*Pa*_ *-Z*_*Pa*_ *-C2*_*Pa*_ gene deletion, containing chloramphenicol resistance gene	This study
pRK415	Broad host range vector, Tc^r^	[18]
pRK415-*phaC1*	*phaC1* complementation vector derived from pRK415 containing *phaC1*coding sequence	This study
pRK415-*phaC2*	*phaC2* complementation vector derived from pRK415 containing *phaC2* coding sequence	This study
pRK415-*phaC2AB*	*phaC2*, *phaA* and *phaB* coding sequence cloned into pRK415	This study
pET28a	Expression vector, Kan^r^, C/N-terminal His-Tag/thrombin/T7-Tag, T7 *lac* promoter, T7 transcription start, f1 origin, *lacI*	Novagen
pET-*phaC1*	Expression vector for PhaC1 with C-terminal His-tag	This study
pET-*phaC2*	Expression vector for PhaC2 with C-terminal His-tag	This study
pET-*phaC1*_*Pa*_	Expression vector for PhaC1_*Pa*_ with C-terminal His-tag	This study

^a^ Cm^r^, Spec^r^, Tc^r^, Kan^r^: resistant to chloramphenicol, spectinomycin, tetracycline and kanamycin, respectively.

### Generation of *phaC* mutants and their complemented strains

All *phaC* mutants were generated by conjugation using a modified mobility plasmid for the transfer between *E*. *coli* WM3064 and *C*. *pinatubonensis* or *Pseudomonas aeruginosa* PAO1 as described previously [19]. The mobility plasmids pEX18Tc-*phaC1*cm, pEX18Tc-*phaC2*spec and pEx18Tc-*phaC*_*Pa*_cm were constructed by fusing PCR products of chloramphenicol resistance gene *cat* from pEX18Tc-*cmgfp* [16] or spectinomycin resistance gene *aadA* from pML300 [17] into the upstream and downstream fragments of targeting genes. These plasmids were transformed into *E*. *coli* WM3064 (2,6-diaminopimelic acid auxotroph) respectively before transferred into *C*. *pinatubonensis* or *Pseudomonas aeruginosa* PAO1 as described previously [19]. The double-crossover recombinants were screened on LB or MSM agar plates containing 0.5 mM MNP and 34 μg/mL chloramphenicol or 100 μg/mL spectinomycin to get desired mutants. The double mutant strain (JMP134Δ*phaC1*Δ*phaC2*) was constructed by deleting *phaC2* in the *C*. *pinatubonensis phaC1* mutant strain (JMP134Δ*phaC1*). The mutant strains were further confirmed by sequencing the PCR product generated from the modified region. All primers are listed in Table 2.

**Table 2:** Oligonucleotides used in this study.

Oligonucleotides	Sequences(5’-3’)
*phaC1*upFor	GATGAATTCCTTCGAAGTCGGTGCCGAAGTC
*phaC1*upRev	CACACATTATCCCAGGTGTCTGATGTAATTGTCTCTCTGCCG
*phaC1*downFor	CATCACGAGATTTCGATTCCCTAAGCTATTCATTGAGAGGACTC
*phaC1*downRev	GAAGGTACCCATTGGCGTAGGCCTTGATCGTG
*cat*For	CGGCAGAGAGACAATTACATCAGACACCTGGGATAATGTGTG
*cat*Rev	GAGTCCTCTCAATGAATAGCTTAGGGAATCGAAATCTCGTGATG
*phaC1*outside	GAGGACGACTGGGACGAAGAGTG
*cat*5’rev	CAACGGTGGTATATCCAGTG
*phaC2*upFor	GCTCTAGACGTCGAATGCCGATCATCTTG
*phaC2*upRev	CACCAAGGTAGTCGGCAAATAACTATGTGACCGAGGCGTAAG
*phaC2*downFor	CGAGGCATAGACTGTACCGTTCGTACCTTGTTCGATC
*phaC2*downRev	CCCAAGCTTCATCGACAAGACGCTCGAG
*aadA*For	CTTACGCCTCGGTCACATAGTTATTTGCCGACTACCTTGGTG
*aadA*Rev	GATCGAACAAGGTACGAACGGTACAGTCTATGCCTCG
*phaC2*outside	CAATATGTCGTTCGCGCTGTG
*aadA*5’Rev	CTGATAGTTGAGTCGATAC
*phaC1*compFor	GTAAAACGACGGCCAGTGAATTCGATGACGATATCGGTCATTTG
*phaC1*compRev	CTCTAGAGGATCCCCGGGTACCATCCTTCGCAGGTAGGCTTG
*phaC2*compFor	GGGGTACCGATGACAACAGCCGTGGGTACG
*phaC2*compRev	GGAATTCTTACGCCTCGGTCACATAGC
*phaC1*qRTFor	AGAACGCTCCGTACCGCTAC
*phaC1*qRTRev	GCATCGACCCATTGCGACAC
*phaC2*qRTFor	ATGACAACAGCCGTGGGTACGAG
*phaC2*qRTRev	ACCATATCTTCGGGGCGCAG
16SqRTFor	AGAGTTTGATCCTGGCTCAG
16SqRTRev	TTCACCCTCAGGTCGTATGCG
*phaC1*expFor	AGAAGGAGATATACCATGGCGACCGGCAAAGGTGC
*phaC1*expRev	TGCGGCCGCAAGCTTTGCCTTGGCCTTGACGTAAC
*phaC2*expFor	CGCGCGGCAGCCATATGATGACAACAGCCGTGGGTAC
*phaC2*expRev	GTGCGGCCGCAAGCTTCGCCTCGGTCACATAGCGTC
*phaC1*_*Pa*_expFor	AGAAGGAGATATACCATGAGTCAGAAGAACAATAACGAGC
*phaC1*_*Pa*_expRev	TGCGGCCGCAAGCTTTCGTTCATGCACGTAGGTTCC
*phaC1*_*Pa*_upFor	GGGGTACCGGCTGGTAGTCGAGCAGTAGC
*phaC1*_*Pa*_upRev	CACACATTATCCCAGGTGTCAGAAGCGCTTGACCGCCGCCGGG
*catPa*For	CCCGGCGGCGGTCAAGCGCTTCTGACACCTGGGATAATGTGTG
*catPa*Rev	GCCCAGGTCCACCGCGGTCCCGCTGAATCGAAATCTCGTGATG
*phaC2*_*Pa*_downFor	CATCACGAGATTTCGATTCAGCGGGACCGCGGTGGACCTGGGC
*phaC2*_*Pa*_downRev	GGGGTACCCATTCGAGGATTCGGTCGCGT
*phaC1*_*Pa*_outside	GAGGTAACACCGAAGAACATC
*phaC2*KpnIFor	CGGGTACCGATGACAACAGCCGTGGGTACG
*phaC2*Rev	CTCTCAATGAATAGCTTAGGTTACGCCTCGGTCACATAGC
*phaAB*For	GCTATGTGACCGAGGCGTAACCTAAGCTATTCATTGAGAG
*phaAB*Rev	GAATTCCATTGTCAATCAGCCCATGTG
RTZPfor	AACGTCGAAGAGGCAAAAGGG
RTZPrev	CGGGTCTGCATGATATGTCC
RTPC2for	CGCTATGTGACCGAGGCGTAAG
RTPC2rev	GACGAAGTCGAACAGCTTGCG
RTC2Jfor	ACGCAGTGCACGGTGGAAATCG
RTC2Jrev	GCGCTGTCAACAATTGCCTCCG
RTJEfor	AGCCGTCATCAGCACGACTG
RTJErev	GTTCCTTGCCAACGAACGCGC

Complementation experiments were performed by first cloning the complete coding sequences of *phaC1* and *phaC2* (amplified by PCR with primers *phaC1*For/*phaC1*Rev and *phaC2*For/*phaC2*Rev, respectively) into the broad host range vector pRK415 [18] via EcoR1 and Kpn1 restriction sites. The resulting plasmids were designated pRK415-*phaC1* and pRK415-*phaC2*, respectively, and were transformed into *E*. *coli* WM3064 and conjugated with *phaC1* and *phaC2* mutant strains respectively to obtain the complemented strains JMP134Δ*phaC1*[pRK415-*phaC1*] and JMP134Δ*phaC2*[pRK415-*phaC2*]. For complementation of the double mutant strain JMP134Δ*phaC1*Δ*phaC2*, both *phaC1* and *phaC2* genes were amplified by PCR and cloned into pRK415 via EcoR1 and Kpn1 sites. The resulting plasmid was transformed into the double mutant strain. The *phaC2* gene along with *phaA* and *phaB* from *C*. *pinatubonensis* JMP134 were amplified and cloned into pRK415, and the resulting plasmid was transformed into *P*. *aeruginosa* PAO1 *phaC* mutant strain.

### Bioinformatics and *in silico* analyses of PHA synthases

Clustal Omega was used to perform multiple-sequence alignment of the reported PHA synthases of known or unconfirmed functions as well as PhaC1 and PhaC2 from *C*. *pinatubonensis* JMP134 [20]. The phylogenetic analysis was performed using the maximum likelihood method with bootstrap analysis (1000 iterations) as described by Quelas, J. I. et al [21]. The tree was constructed using MEGA (version 5.1) software [22]. The occurrence of conserved residues in the catalytic triad and the lipase box was checked manually.

### Real-time quantitative PCR

The bacterial strains were grown in minimal medium with 0.5 mM MNP or 0.2% octanoate as the sole carbon source to the early stationary phase (approximately 12 hours after inoculation). Total RNA was isolated with an RNA prep pure bacterial kit (Tiangen Biotech, Beijing, China) and reversely transcribed into cDNA using a PrimeScript RT Reagent Kit with gDNA Eraser (Perfect Real Time) (TaKaRa, Dalian, China). Real-time quantitative PCR (RT-qPCR) was performed on a CFXConnect^™^ Real-Time PCR Detection System (Bio-Rad, Hercules, CA, USA) using iQ SYBR Green Supermix (Bio-Rad, Hercules, CA, USA) with primers specific to individual *phaC* genes or 16S rRNA gene listed in Table 2. The amount of target mRNA was normalized to that of 16S rRNA gene with the 2^-ΔΔC^_T_ method as described previously [19].

### PHA monomer determination and quantification

The intracellular PHA contents and PHA composition were determined by gas chromatography-mass spectrometry (GC-MS) as described previously [23, 24]. Briefly, bacterial cells cultured for 48 hours (late stationary phase) were harvested by centrifugation at 15,000 × *g* for 15 min, washed with phosphate buffered saline (PBS) (pH 7.4, 20 mM) and lyophilized overnight. Lyophilized bacterial cells (15 mg) were subjected to methanolysis in the presence of 3% H_2_SO_4_ (v/v) at 95°C for 4 hours, washed with ddH_2_O and extracted with chloroform. The resulting methyl esters of the constituent 3-hydroxyalkanoic acids were diluted and analyzed by GC-MS according to the literature [23]. The GC analysis was performed by injecting 50 μl of each sample into a TRACE GC Ultra gas chromatography (Thermo Fisher scientific Inc., MA, USA) using a capillary column TRACE TR-1701 (0.25 mm × 30 meters, Thermo Fisher scientific Inc., MA, USA). The mass spectrometry was performed as described previously [25]. Polyhydroxybutyrate (PHB) (Sigma-Aldrich Co., St. Louis, MO, USA) or 3-hydroxyoctanoate (3-HO) (Sigma-Aldrich Co., St. Louis, MO, USA) was used as a standard, and benzoic acid was used as an internal standard for PHA quantification. Under these conditions, authentic PHB, 3HO, and benzoic acid had retention times of 6.02 min, 11.76 min, and 9.18 min, respectively. The methyl esters can be used to indicate methanolysized PHA and were identified with a NIST98 MS data library based on the comparison of both the GC retention time and the mass spectra with those of the authentic compounds.

### Protein expression and purification

The PHA synthase genes *phaC1* and *phaC2* of strain JMP134 and *phaC1* (PA5056) from *P*. *aeruginosa* PAO1 were amplified using primers (Table 2) and then cloned into pET28a via NcoI and HindIII restriction sites to obtain pET-*phaC1*, pET-*phaC2* and pET-*phaC1*_*Pa*_, respectively. *E*. *coli* BL21(DE3) strains carrying the resulting expression constructs were grown at 37°C to an OD_600_ of 0.4~0.6 and then induced with 0.1 mM isopropyl-D-thiogalactopyranoside (IPTG) at 30°C for 5 hours or at 16°C for overnight. Bacterial cells were collected by centrifugation at 15,000 × *g* for 10 min and lysed by sonication. Cell crude extracts were centrifuged at 12,000 × *g* for 30 min to remove the cell debris, and the supernatants were applied to a Ni^2+^-NTA (nickel-nitrilotriacetic acid) agarose chromatography column (Novagen, Madison, WI) to purify the C-terminal His-tagged PhaC1-H_6_, PhaC2-H_6_ and PhaC1_*Pa*_-H_6_. The non-specific binding proteins were washed by phosphate buffered saline (PBS) containing 80 mM imidazole and the His-tagged proteins were eluted with PBS containing 200 mM imidazole as described [19]. Proteins were analyzed by SDS-PAGE and stored at 4°C.

### PHA synthase activity determination

The standard assay (final volume of 1 mL) used 0.5 mM dithionitrobenzoic acid (DTNB), 20 mM MgCl_2_ and 50 μM of the 3-hydroxybutyryl-CoA (3-HBCoA) (Sigma Chemical Co., St. Louis, MO, USA) or 3-hydroxyoctanoyl-CoA (3-HOCoA) (custom synthesized by Shanghai SIMR Biotech Co., Ltd, Shanghai, China) in 50 mM PBS, pH 7.5. Both 3-HBCoA and 3-HOCoA are mixtures of their *R*- and *S*-stereoisomers (50% each) and only the *R*-stereoisomers were calculated in the study since PHA synthases can only utilize the *R*-stereoisomer of hydroxyacyl-CoA [4]. The PHA synthase activity was determined spectrophotometrically by monitoring the product formed between DTNB and the coenzyme A (CoA) released from the substrate 3-HBCoA or 3-HOCoA at 412 nm with a molar absorption coefficient of 13,600 M^-1^cm^-1^ as described by Valentin et al. [26]. One unit of enzyme is defined as the amount needed to produce 1 μmol product per minute under these assay conditions employed. The protein concentration was determined using the Bradford reagent (Sigma-Aldrich Co., St. Louis, MO, USA).

### Measurement of bacterial growth on MNP

The growth of strain JMP134 and its derivatives in 0.5 mM MNP was measured at OD_600_. The growth curves to the time were fitted by the modified Gompertz equation with OriginPro 8.0 software as described previously [27, 28]. Their maximum specific growth rates (μ_max_) were calculated.

## Results

### *In silico* analysis revealed that *phaC1* and *phaC2* encode two distinct classes of PHA synthases

**Fig 1:**
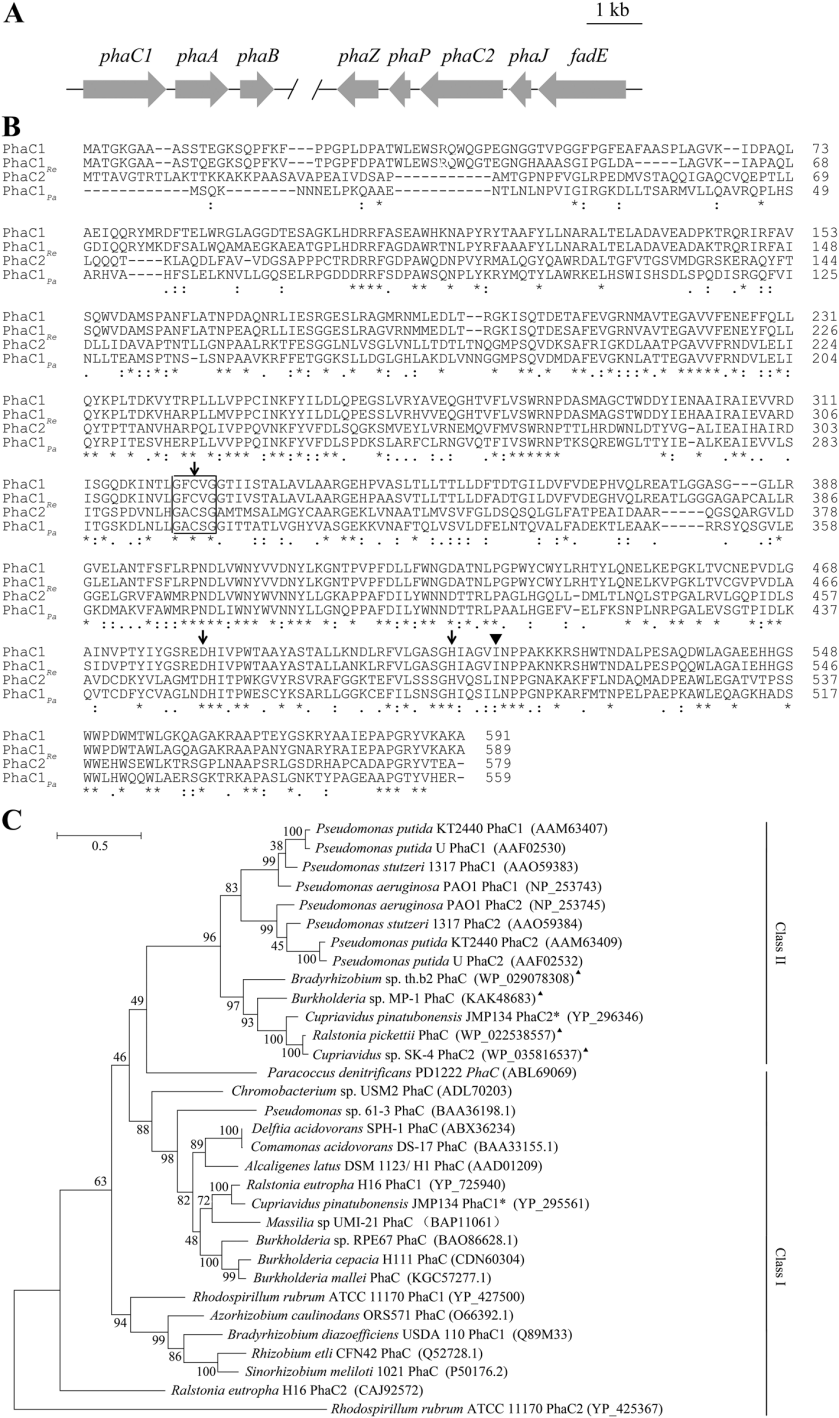
The gene clusters containing *phaC1* and *phaC2* in *C*. *pinatubonensis* JMP134 and *in silico* analysis of their products. (A) Organization of gene clusters containing *phaC1* and *phaC2* obtained from the reported genome sequence. The gene cluster containing *phaC1* begins at nucleotide position 1447474 and ends at 1451315 on the Watson strand in the genome of *C*. *pinatubonensis* JMP134 (NCBI Reference Sequence: NC_007347.1). The gene cluster containing *phaC2* begins at nucleotide position 2356098 and ends at 2361552 on the Crick strand in the genome. Open reading frames were annotated with a guide by BLAST results. PhaA: *β*-ketothiolase; PhaB: acetoacetyl-CoA reductase; PhaZ: PHA depolymerase; PhaP: phasin; PhaJ: *R*-specific enoyl-CoA hydratase; FadE: acyl-CoA dehydrogenase. (B) Amino acid sequence alignment of PhaC1 and PhaC2 from *C*. *pinatubonensis* JMP134, a typical class I PHA synthase PhaC1_*Re*_ from *R*. *eutropha* H16 and a typical class II PHA synthase PhaC1_*Pa*_ from *P*. *aeruginosa* PAO1. The boxed region indicates the signature motif of lipase sequence. The arrows indicate conserved residues involved in catalysis (catalytic triad). The triangle indicates a critical residue affecting substrate specificity. (C) Phylogenetic tree constructed using a multiple sequence alignment of several PHA synthases. Classes of different PHA synthases are indicated on the right side of the tree. The triangles indicate PHA synthases of unconfirmed function. The asterisks indicate PHA synthases from strain JMP134. All protein accession numbers are given in brackets.

The *C*. *pinatubonensis* JMP134 genome contains two genes—*phaC1* and *phaC2*—predicted to encode two PHA synthases according to the sequence analysis. As shown in Fig 1A, these two genes are in two distinct PHA synthesis clusters at different loci in the genome. The *phaC1* gene is located in front of the putative genes encoding acetyl-CoA acetyltransferase (*phaA*) and acetoacetyl-CoA reductase (*phaB*). These are highly similar to the genes involved in the synthesis of polyhydroxybutyrate (PHB) in *Ralstonia eutropha* H16. The *phaC2* gene is located in a five-gene cluster that contains genes encoding the putative acyl-CoA dehydrogenase (*fadE*), MaoC-like dehydratase (*phaJ*), PHA granule-associated protein (*phaP*) and PHA depolymerase (*phaZ*)—all of these are predicted to be involved in PHA metabolism. PhaC1 and PhaC2 contain 591 and 579 amino acids respectively, with 39% sequence identity. Their major differences come from the N-terminus of the proteins. The C-terminus has high sequence similarity including the catalytic triad Cys296, Asp451 and His479, as well as the conserved lipase box sequence GXCXG (Fig 1B), which are common features in PHA synthases.

A phylogenetic tree was constructed based on a multiple-sequence alignment of PHA synthase protein sequences with known or unconfirmed functions using a maximum likelihood method with 1000 bootstraps (Fig 1C). PhaC1, closely related to the well-studied PhaC1 of *Ralstonia eutropha* H16, was classified as a class I PHA synthase. The PhaC2 was grouped with class II PHA synthases based on the phylogenetic tree. Interestingly, PhaC2 showed a moderate sequence identity (approximately 50%) to the class II PHA synthases of known functions from *Pseudomonas* spp. strains. But its closer homologs were several putative class II PHA synthases genes of unconfirmed functions. Of the clusters containing functionally identified class II PHA synthase genes so far, two different class II PHA synthase genes were present and separated by the *phaZ* gene encoding an intracellular PHA depolymerase [3, 29, 30]. In contrast, the *phaC2* gene cluster in strain JMP134 has a distinct gene organization without the additional synthase gene (Fig 1A).

### Both PhaC1 and PhaC2 were functional for PHA synthesis *in vivo*

To investigate if the two *phaC* genes are involved in PHA accumulation, the *phaC1*, *phaC2* or both genes were deleted from the strain JMP134 genome by allelic replacement to generate JMP134Δ*phaC1*, JMP134Δ*phaC2* and JMP134Δ*phaC1*Δ*phaC2* mutant strains, respectively. PHA production from these strains when grown in 0.2% (w/v) fructose or octanoate was monitored with GC-MS. Here, the only hydroxyalkanoic methyl ester detected in samples of strain JMP134 grown in MNP, fructose or octanoate was 3-hydroxybutyric methyl ester, indicating that strain JMP134 can only produce PHB. In samples of Δ*phaC1* and Δ*phaC2* strains when grown in fructose or octanoate, 3-hydroxybutyric methyl ester was also detected, suggesting that the Δ*phaC1* and Δ*phaC2* mutant strains could still produce PHB as the wild type strain (Table 3). In contrast, no hydroxyalkanoic methyl ester was detected in the double mutant strain JMP134Δ*phaC1*Δ*phaC2*, suggesting that there are no additional functional PHA synthases in this strain (Table 3). The 3-hydroxybutyric methyl ester was detected in the complemented strain of the JMP134Δ*phaC1*Δ*phaC2* double mutant with either *phaC1* or *phaC2* gene. This suggests that both PhaC1 and PhaC2 are functional PHA synthases. Furthermore, the quantification analysis showed that no significant difference in PHB content of strain JMP134 grown in 0.2% (w/v) fructose or octanoate (Table 3). In addition, both JMP134Δ*phaC1* and JMP134Δ*phaC2* strains accumulated similar amounts of PHB as JMP134 when grown in these carbon sources (Table 3). This suggests that different carbon sources did not affect the amount of PHB produced in these strains regardless of the presence of functional PhaC1 or PhaC2 or both.

**Table 3:** PHA contents in the lyophylized samples of *C*. *pinatubonensis* JMP134, *phaC* mutant strains and their complemented strains.

	PHA content (% of cell dry weight)^b^		
Strains^a^	MNP	Fructose	Octanoate
JMP134	44.57±7.71	49.40±8.73	50.87±5.13
JMP134Δ*phaC1*	0.33±0.37	43.22±10.88	55.8±3.67
JMP134Δ*phaC2*	43.22±16.38	53.07±4.37	56.33±3.33
JMP134Δ*phaC1*Δ*phaC2*	0.72±0.53	0.50±1.33	1.02±0.30
JMP134Δ*phaC1*[pRK415-*phaC1*]	46.62±5.2	48.58±5.06	52.66±7.01
JMP134Δ*phaC2*[pRK415-*phaC2*]	40.91±12.17	46.24±8.03	50.78±8.67
JMP134Δ*phaC1*Δ*phaC2* [pRK415-*phaC1-phaC2*]	41.34±10.33	43.37±6.67	54.66±4.47
JMP134Δ*phaC1*Δ*phaC2* [pRK415-*phaC1*]	44.79±7.43	51.28±8.34	54.55±11.34
JMP134Δ*phaC1*Δ*phaC2* [pRK415-*phaC2*]	40.27±4.42	46.72±6.32	50.88±9.73

^a^ All bacterial strains were cultured at 30°C for 48 hours and shaken at 250 rpm with 0.5 mM MNP, 0.2% fructose or 0.2% octanoate, respectively, before lyophylization.

^b^ The PHA contents of the lyophylized samples (% of cell dry weight) were analyzed and quantified by GC-MS. The results are the average of three replicate experiments.

Interestingly, with 0.5 mM MNP (0.0068% w/v) as the sole carbon source, PHA was detected in both JMP134 and JMP134Δ*phaC2* samples. There was very little PHA accumulated in the mutant strains JMP134Δ*phaC1* and JMP134Δ*phaC1*Δ*phaC2* when grown in MNP. This indicated that PhaC1 but not PhaC2 is involved in the synthesis of PHB when grown in 0.5 mM MNP. Both JMP134 and JMP134Δ*phaC2* strains accumulated similar PHB contents when grown with MNP, comparing to that with fructose or octanoate (Table 3).

### Transcriptional levels of *phaC1* and *phaC2*

**Fig 2:**
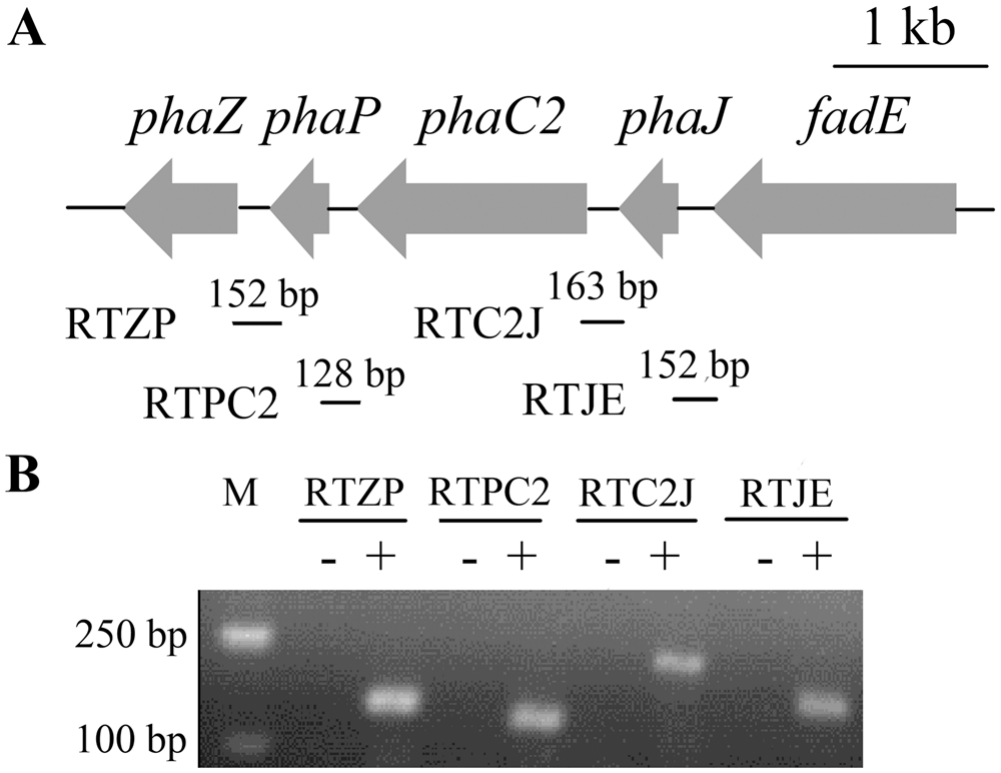
Transcriptional organization analysis of *phaZ*, *phaP*, *phaC2*, *phaJ* and *fadE* genes. Strain JMP134 was grown in MSM supplemented with 2% octanoate (w/v) at 30°C, and mRNA was extracted at the early stationary phase. The cDNA was synthesized with random primers. (A) Schematic of the gene cluster containing *phaC2*. The locations and number of nucleotides of DNA fragments amplified by PCR are represented by short solid lines below the relevant genes and designated RTZP, RTPC2, RTC2J and RTJE. (B) Electrophoresis of PCR products from transcriptional organization analysis of RTZP, RTPC2, RTC2J and RTJE. Lane M, molecular marker; +, presence of RT-PCR products; -, the corresponding negative controls with DNase-treated RNA samples.

To investigate the transcription of *phaC1* and *phaC2* when strain JMP134 was grown on different carbon sources, their transcriptional levels were assessed by qRT-PCR. With 0.5 mM MNP as the sole carbon source, the transcriptional level of *phaC1* was approximately 70-fold (70±9.17) higher than that of *phaC2*. In contrast, when grown in 0.2% octanoate, the transcriptional level of *phaC2* was approximately 240-fold (244±45.21) higher than that of *phaC1*. Interestingly, *phaC2* was co-transcribed with its neighbor genes that are predicted to be involved in fatty acid metabolism (Fig 2). The data indicated that *phaC1* and *phaC2* in strain JMP134 were differentially transcribed when induced with different substrates or metabolites.

### PhaC1 and PhaC2 activities *in vitro*

**Fig 3:**
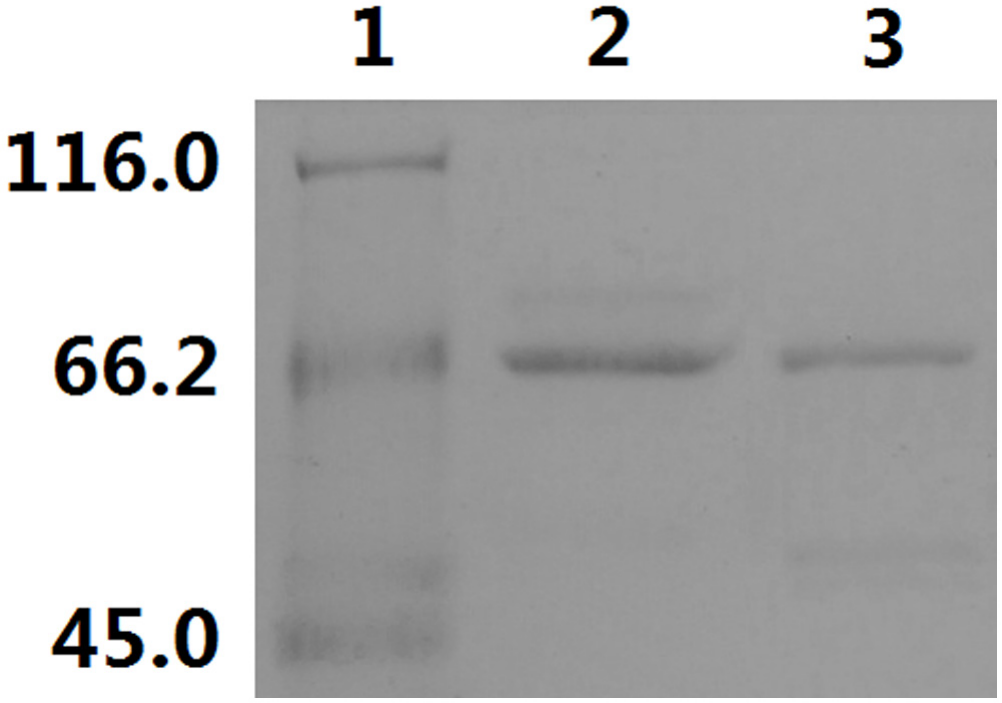
SDS-PAGE of purified PhaC1-His_6_ and PhaC2-His_6_. Lane 1, molecular mass standards (molecular mass are indicated on the left in kDa); lane 2, purified PhaC1-His_6_; and lane 3, purified PhaC2- His_6_.

To investigate whether PhaC1 and PhaC2 exhibit activities towards SCL hydroxyacyl-CoA substrate 3-hydorxybutyryl-CoA (3HBCoA) and MCL hydroxyacyl-CoA substrate 3-hydroxyoctanoyl-CoA (3HOCoA) *in vitro*, these two enzymes were recombinantly produced and purified, with a similar molecular mass of approximately 65 kDa (Fig 3). Both PhaC1 and PhaC2 showed activity towards 3HBCoA but not 3HOCoA. In contrast, the typical class II PHA synthase PhaC1_*Pa*_ from *P*. *aeruginosa* as a control displayed enzyme activity towards 3HOCoA but not 3HBCoA (Table 4). This suggested that both PhaC1 and PhaC2 had the same substrate specificity, only catalyzing the polymerization of SCL substrate 3-hydroxyacyl-CoA rather than MCL substrate 3-hydroxyacyl-CoA. The kinetics parameters of PhaC1 and PhaC2 towards 3HBCoA were listed in Table 4. The *K*_*m*_ value of PhaC1 for 3HBCoA was lower than that of PhaC2, suggesting that PhaC1 has a higher affinity to 3HBCoA than PhaC2.

**Table 4:** Kinetic parameters of recombinant PHA synthases towards 3-hydroxybutyryl-CoA (3HBCoA) and 3-hydroxyoctanoyl-CoA (3HOCoA).

PHA synthase	Substrate	*K*_*m*_ (μM)	*K*_*cat*_ (min^-1^)	*K*_*cat*_/*K*_*m*_ (μM^-1^·min^-1^)	*V*_*max*_ (μM·min^-1^)
PhaC1	3HBCoA	45.46±6.02	39.85±2.11	0.88±0.19	38.32±2.03
	3HOCoA	ND	ND	ND	ND
PhaC2	3HBCoA	115.93±9.15	3.75±0.02	0.032±0.002	1.74±0.10
	3HOCoA	ND	ND	ND	ND
PhaC1_*Pa*_	3HBCoA	ND	ND	ND	ND
	3HOCoA	67.28±12.86	56.39±5.30	0.83±0.25	39.02±3.67

The kinetic constants were calculated by nonlinear regression analysis, and the values are expressed as means ± standard deviations (n = 3). ND, not detectable.

### PhaC2 also heterologously functions as a class I PHA synthase

PhaC2 along with PhaAB from strain JMP134 were heterologously expressed in *P*. *aeruginosa* PAO1 *phaC* mutant strain (PAO1Δ*phaC1*_*Pa*_-*Z*_*Pa*_-*C2*_*Pa*_). The PHA content and composition in strain PAO1 and its derived strains were analyzed after cultivation in 0.2% (w/v) octanoate. The results indicated that strain PAO1 produced 22.8 ± 2.2% 3-hydroxyoctanoate (3HO)-based PHA of cell dry weight but its *phaC* mutant did not produce detectable PHA (Table 5). Interestingly, its mutant strain expressing PhaC2, PhaA, and PhaB from strain JMP134 accumulated 42.2 ± 12.5% 3-hydroxybutyrate (3HB)-based PHA. This suggested that PhaC2 from *C*. *pinatubonensis* still preferred SCL substrates in a *Pseudomonas* spp. as in its native background.

**Table 5:** Content and composition analyses of PHA in *P*. *aeruginosa* PAO1 and its derived strains grown in octanoate^a^.

Strains^b^	PHA content (% of cell dry weight)^c^	Polymer composition (mol%)^d^	
		3-HB	3-HO
PAO1	22.8±2.2	ND^e^	100
PAO1Δ*phaC1*_*Pa*_ *-Z*_*Pa*_ *-C2*_*Pa*_	ND	ND	ND
PAO1Δ*phaC1*_*Pa*_ *-Z*_*Pa*_ *-C2*_*Pa*_[pRK415-*phaC2AB*]	42.2±12.5	100	ND

^a^ Cells were cultivated at 30°C for 48 hours and shaken at 250 rpm with 0.2% (w/v) octanoate. GC-MS analyses of intracellular PHA content and PHA composition were performed. All results were obtained from three different experiments.

^b^ PAO1, *P*. *aeruginosa* PAO1; PAO1Δ*phaC1*_*Pa*_ *-Z*_*Pa*_ *-C2*_*Pa*_, *phaC* mutant strain of *P*. *aeruginosa* PAO1 with *phaC1*_*Pa*_ *-Z*_*Pa*_-*C2*_*Pa*_ deleted; PAO1Δ*phaC1*_*Pa*_ *-Z*_*Pa*_ *-C2*_*Pa*_[pRK415-*phaC2AB*], the *phaC* mutant of strain PAO1 transformed with plasmid pRK415 expressing PhaC2, PhaA, and PhaB from *C*. *pinatubonensis* JMP134.

^c^ PHA content is presented as percentage of the cell dry weight.

^d^ 3-HB, 3-hydroxybutyrate; 3-HO, 3-hydroxyoctanoate.

^e^ ND, not detectable.

### Bacterial growth of strain JMP134 and its *phaC* mutant strains

**Fig 4:**
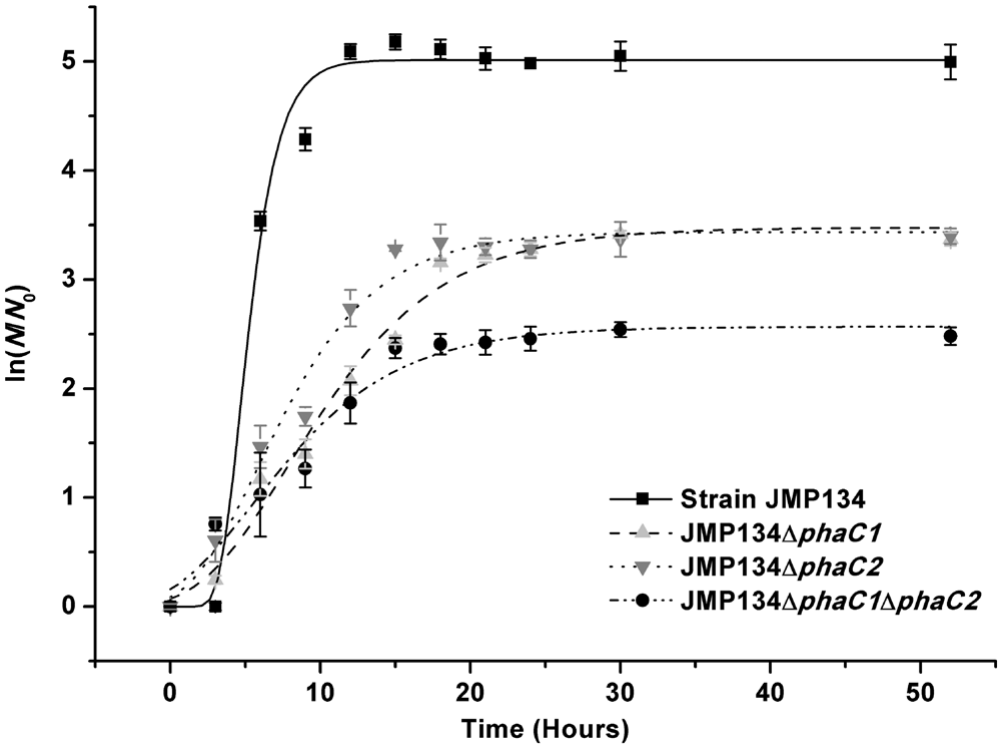
Growth kinetics of *C*. *pinatubonensis* JMP134 and derived *phaC* mutant strains on MNP. All strains were grown in minimal salt medium containing 0.5 mM MNP. Their growth was evaluated as measurements of the optical density at 600 nm. N represents the OD_600_ value; N_0_ represents the initial OD_600_ values. All points represent the mean values of triplicate trials with error bars denoting their standard deviations.

The optical density at 600 nm of strains JMP134, JMP134Δ*phaC1*, JMP134Δ*phaC2*, and JMP134Δ*phaC1*Δ*phaC2* cultured in MSM with 0.5 mM MNP were assessed every 3 hours to investigate if the formation of intracellular PHA would affect bacterial growth. All strains could grow in media containing MNP as the sole carbon source (Fig 4). The maximum growth rate of strain JMP134 (0.78 h^-1^) in MNP was higher than those of JMP134Δ*phaC1* (0.22 h^-1^), JMP134Δ*phaC2* (0.29 h^-1^), and JMP134Δ*phaC1*Δ*phaC2* (0.18 h^-1^) strains indicating a correlation between the PHA accumulation (stated in Table 3) and the growth.

## Discussion

Of the PHA synthase genes with known functions in PHA-producing bacteria, *phaC* paralogs tend to come from a same PHA synthase class if two or more such gene copies are present in the genome [3, 31]. In this study, *phaC1* and *phaC2* in the versatile aromatic degrader *C*. *pinatubonensis* JMP134 encode functional PHA synthases with same substrate preference but belong to PHA synthase class I and class II, respectively. Subsequent biochemical and genetic analyses demonstrated that, although their products have the same substrate preference, these genes coding for the two PHA synthase were transcribed differentially when different carbon sources were utilized by this strain.

It is generally accepted that the class I and class II PHA synthases prefer SCL and MCL substrates, respectively [3]. In this study, biochemical and genetic evidence has demonstrated that, although being categorized in class II, the PhaC2 displayed a distinct substrate preference comparing to other reported class II PHA synthases regardless of the carbon sources used (fructose or octanoate) or metabolic backgrounds (*C*. *pinatubonensis* JMP134 or *P*. *aeruginosa* PAO1). Furthermore, *in vitro* enzyme activity assay demonstrated that PhaC2 exhibited activity towards SCL substrate 3HBCoA but not MCL substrate 3HOCoA, exhibiting a typical substrate specificity of class I PHA synthase. So far, most class II PHA synthases only showed activity towards MCL substrates [3]. Limited cases have described class II PHA synthase with a broad substrate range. For example, PhaC1 and PhaC2 from *Pseudomonas* sp. 61–3 [32] as well as PhaC2 from *Pseudomonas stutzeri* 1317 [33] were shown to catalyze the copolymerization of both SCL and MCL substrates. However, in this study, PhaC2 from strain JMP134 catalyzed the polymerization of SCL substrate (3-hydroxybutyryl-CoA) only—but no MCL PHA from substrate (3-hydroxyoctanoyl-CoA) was produced. This indicates a distinct substrate binding spectrum of this enzyme among the class II PHA synthases.

Several studies have revealed the relationship between the functions of different types of PHA synthases and their critical residues [34, 35]. Substituting of leucine at amino acid residue 484 (most conserved residue at the corresponding position in class II PHA synthases) to valine in class II PHA synthase from *Pseudomonas putida* Gpo1 altered its substrate specificity from SCL only to both SCL and MCL [36] (valine and isoleucine are the most conserved residues at the corresponding position in class I and III PHA synthases). Interestingly, an isoleucine at residue 484 was also found in PhaC2. This may be responsible for its unique substrate specificity in this case. This substrate preference of PhaC2 from strain JMP134 is different from those of class II PHA synthase (PhaC1 and PhaC2 from *Pseudomonas* sp. 61–3 or PhaC2 from *Pseudomonas stutzeri* 1317), indicating a requirement for new parameters in PHA synthase classification and prediction.

Despite having the same substrate preference to synthesize PHB *in vitro*, the genes encoding PhaC1 and PhaC2 are regulated differently in strain JMP134 as illustrated by qRT-PCR analysis of their transcriptional levels. The higher transcriptional level of *phaC1* in MNP-grown cells indicates that intermediates of MNP catabolism may be involved in the triggering of *phaC1* transcription. This correlates with the sole involvement of PhaC1 in PHB synthesis of MNP-grown cells and suggests its role in PHA synthesis when utilizing an unfavorable carbon source (MNP). In contrast, the higher transcriptional level of *phaC2* was shown in cells grown in octanoate—a favorable carbon source for this bacterium. Generally, the regulation of PHA synthesis genes is affected by the growth stage and nitrogen limitation [3]. The observation of differential transcription of *phaC* paralogs when utilizing different carbon sources is of interest but has not yet been reported. These obvious variations in transcription on *phaC1* and *phaC2* may well reflect the differences in their transcriptional regulations towards unfavorable carbon sources or readily usable carbon sources.

In the PHA synthesis involving class II PHA synthase, the encoding gene clusters exhibit a common feature that two different *phaC* genes in the same class are separated by *phaZ* encoding for an intracellular depolymerase. Genes encoding enzymes related to the biosynthesis of PHA precursors are normally scattered in the genomes of PHA producers [3]. In contrast, the cluster in strain JMP134 has a single copy of the class II PHA synthase-encoding gene. This gene is clustered and co-transcribed with genes encoding enzymes predicted to be involved in the synthesis of MCL 3-hydroxyalkanoate. Considering the PhaC2 substrate preferences, we speculated that this gene cluster was evolved from a cluster encoding a set of enzymes responsible for synthesizing MCL-PHA. This unique *phaC2*-containing genetic and transcriptional organization may well present a different evolutionary path among PHA producers with class II PHA synthases.
